# A cross-sectional study of mental health and well-being among veterinarians in Pakistan

**DOI:** 10.3389/fvets.2026.1845572

**Published:** 2026-07-15

**Authors:** M. Yasir Zahoor, Muhammad Bilal, Kanza Arshad, Shoaib Ashraf

**Affiliations:** 1 Department of Comparative Medicine and Integrative Biology, College of Veterinary Medicine, Michigan State University, East Lansing, MI, United States; 2 Faculty of Veterinary Medicine, University of Calgary, Calgary, AB, Canada; 3 Institute of Biochemistry and Biotechnology, University of Veterinary and Animal Sciences, Lahore, Pakistan; 4 Department of Biomedical Sciences, Ross University School of Veterinary Medicine, Basseterre, Saint Kitts and Nevis

**Keywords:** anxiety, depression, mental health, mental health survey, veterinarians

## Abstract

Veterinarian mental health is an important occupational and public health concern. However, limited data are available from Pakistan. This study aimed to assess mental health, professional quality of life, and suicidal ideation among veterinarians in Pakistan. A total of 456 veterinarians participated in a web-based structured questionnaire that included demographic and occupational characteristics, the Hospital Anxiety and Depression Scale (HADS-A and HADS-D), the Professional Quality of Life Scale (ProQOL-5), and suicidal ideation-related items. The overall median HADS-A score was 9.0 (IQR: 6–16), with 42.1% classified in the abnormal anxiety category. The overall median HADS-D score was 9.0 (IQR: 5–12), with 38.4% classified in the abnormal depression category. Comorbid abnormal anxiety and depression were observed in 30.7% of participants. Female veterinarians had higher median anxiety, depression, and secondary traumatic stress scores than male veterinarians. Compassion satisfaction was negatively correlated with burnout, anxiety, and depression, whereas burnout and secondary traumatic stress were positively correlated with anxiety and depression. In multivariable logistic regression, female participants had higher odds of reporting suicidal ideation. This study provides baseline evidence of substantial mental health and professional well-being concerns among veterinarians in Pakistan. The findings highlight the need for targeted mental health support, workplace-based interventions, and policy-level strategies to improve the well-being and sustainability of the veterinary workforce.

## 1 Introduction

Mental health, as defined by the World Health Organization (WHO), is a “state of well-being in which an individual realizes his or her abilities, can cope with the normal stresses of life, can work productively and can contribute to his or her community” (1). Veterinarians have been reported to experience higher levels of psychological distress and suicidal tendencies than the general public (2, 3). Several surveys have supported these findings, with some studies indicating that suicidal risk is three to four times higher among veterinarians (4, 5). In Germany, one survey classified 32% of veterinarians as being at high risk of suicide compared with less than 7% of the general public (6). In the United States, proportional mortality ratios for suicide have been reported to be higher among veterinarians than in the general population, particularly among female veterinarians (7).

In Pakistan, veterinary practice is regulated by the Pakistan Veterinary Medical Council (PVMC), which is responsible for the registration and regulation of veterinary medical practitioners^1^. Publicly available national statistics from the Pakistan Bureau of Statistics, sourced from PVMC, reported 17,257 registered veterinary medical practitioners up to December 31, 2021 (8). Veterinarians in Pakistan work across diverse professional settings, including public-sector livestock and animal health services, private clinical practice, poultry and dairy production systems, diagnostic laboratories, academia, research institutions, and field-based animal health programs. These roles often involve long working hours, direct animal handling, client interactions, exposure to zoonotic diseases, emergency duties, and resource-limited working conditions, all of which may contribute to occupational stress and mental health risks among veterinarians (9). Therefore, understanding mental health and occupational well-being in this professional group is important not only for individual veterinarians but also for animal health services, public health, food security, and One Health systems in Pakistan (10, 11).

Various occupational stressors, such as dependence on clients for livelihood (12), a demanding job with low autonomy (13), low reward despite high efforts (14), and the demands of clinical practice, are relevant, although not unique, to the veterinary profession (13). Burnout is an important occupational construct in veterinary medicine. According to the ICD-11, burnout is not classified as a medical condition but as an occupational phenomenon resulting from chronic workplace stress that has not been successfully managed (15, 16). It is characterized by three dimensions: feelings of energy depletion or exhaustion, increased mental distance from one’s job or feelings of negativism or cynicism related to work, and reduced professional efficacy. This definition is important because it frames burnout as a consequence of persistent occupational demands and workplace conditions rather than as a personal weakness or individual failure (16, 17). Recently, several studies have also indicated increasing concerns regarding mental well-being (MWB) among veterinarians during and after the COVID-19 pandemic (18).

During the early stages of a veterinary career, emotional stressors, challenging working conditions, and business-related pressures may contribute to disillusionment, reduced professional enthusiasm, and burnout, potentially leading to significant mental and physical health consequences (19). Beyond its effects on the individual, burnout can also affect the provision of quality care to patients (15, 16, 20). Because veterinarians must interact with both the animal patients and their owners, burnout may influence animal care, client communication, and professional performance. The main components of burnout include exhaustion and fatigue resulting from interaction between individuals and workplace stress (21). Burnout experiences vary among individuals and may include feelings of frustration, being overwhelmed, lack of enthusiasm, being overworked, and feeling trapped (22). Core themes include distressing emotions and poor compatibility between the individual and the work environment. Well-being has been reported to be affected by six major areas of mismatches: work overload, lack of control, insufficient reward, community breakdown, lack of fairness, and conflicting values in the workplace (23). Emergency care settings are also associated with considerable psychological demands, where professionals may experience fatigue due to difficult working conditions, insufficient sleep, limited resources, and poor support. These factors can lead to irritability and health-related consequences that negatively affect the quality of life (24). To address these challenges, many veterinary organizations have initiated awareness campaigns and mental well-being support programs; however, such programs are not universally available to all veterinarians (18).

In addition to burnout, several related constructs are relevant to veterinary mental health (25, 26). Secondary traumatic stress refers to work-related distress that can occur through indirect exposure to the traumatic experiences, suffering, injury, or death of others (27). Compassion fatigue is commonly used to describe the negative emotional and psychological effects of caring for those experiencing suffering or trauma and is often conceptualized as including burnout and secondary traumatic stress (25, 26). In contrast, compassion satisfaction reflects the positive aspect of professional quality of life, including the sense of meaning, fulfillment, and pleasure derived from helping others and performing one’s work effectively (28). Suicidal ideation refers to thinking about, considering, or planning suicide and is a critical indicator of mental health risk (29, 30). These constructs are particularly relevant to veterinarians, who may regularly encounter animal suffering, euthanasia, client distress, economic constraints in care, occupational hazards, and emotionally demanding work environments (31, 32).

To our knowledge, this is among the first studies to examine mental health, burnout, compassion-related professional quality of life, and suicidal ideation among veterinarians in Pakistan. These data are essential for informing the development of targeted, evidence-based prevention and intervention strategies, as well as guiding policymakers and professional bodies in implementing supportive frameworks to improve the mental health and overall well-being of veterinarians in Pakistan.

## 2 Materials and methods

A cross-sectional survey was conducted online using Microsoft Forms. A structured questionnaire was administered to veterinarians in Pakistan from August 2024 to November 2024. Before accessing the questionnaire, participants were provided with information about the study purpose, voluntary participation, confidentiality, and their right to withdraw before submission. Written informed consent was obtained electronically from all participants before they proceeded to the questionnaire.

### 2.1 Participant recruitment

Participants in the study were recruited from across Pakistan to obtain broad geographical representation. Eligible participants were required to be veterinarians currently residing or practicing in Pakistan, able to read and write English, and 20 years of age or older. The final questionnaire link was sent through multiple channels, including emails, text messages, and digital posters shared on online platforms and professional networks. Participation was voluntary, and respondents completed the questionnaire electronically using the provided survey link. Data collection was conducted over a four-month period, during which all responses were received and recorded for further analysis.

### 2.2 Questionnaire

The questionnaire consisted of 4 sections: sociodemographic and occupational characteristics, the Hospital Anxiety and Depression Scale Anxiety and Depression Subscales (HADS-A & HADS-D), the Professional Quality of Life Scale, version 5 (ProQOL-5), and suicidal ideation. Demographic and occupational questions included gender, age, marital status, employment status, type of work, and working hours. The “other” category for main type of work included professional roles not captured by the listed options, such as zoo veterinarians, wildlife veterinarians, laboratory-based veterinarians, and other non-traditional veterinary roles. Salary was not included because income is a sensitive topic in Pakistan and its inclusion could have reduced response rates. Furthermore, the diverse employment settings of veterinarians result in highly variable income structures that may not accurately reflect socioeconomic status.

Anxiety and depression were assessed using the Hospital Anxiety and Depression Scale (HADS). Suicidal ideation was assessed using three items adapted from the National Survey of Psychiatric Morbidity/Adult Psychiatric Morbidity Survey framework, which has been used in population-based psychiatric morbidity research to assess suicidal thoughts and related outcomes (33, 34). Professional quality of life was assessed using the Professional Quality of Life Scale (ProQOL-5), which includes three subscales: compassion satisfaction, burnout, and secondary traumatic stress (25, 35).

The questionnaire was administered in English. This was considered appropriate because veterinary education and professional documentation in Pakistan are commonly conducted in English. However, because the HADS, ProQOL-5, and suicidal ideation items have not been specifically validated among Pakistani veterinarians, the results should be interpreted as screening and descriptive indicators rather than clinical diagnoses.

### 2.3 Hospital Anxiety and Depression Scale (HADS)

Anxiety and depression among veterinarians were assessed using the HADS, originally developed by Snaith and Zigmond (36), which is a 14-item self-report scale consisting of two 7-item subscales: HADS-A and HADS-D. Each item is scored from 0 to 3, giving a possible score range of 0–21 for each subscale. Score categories were interpreted according to standard HADS scoring guidance: 0–7 as normal, 8–10 as borderline abnormal, and 11–21 as abnormal (37). The HADS has been widely used as a screening measure for anxiety and depression symptoms in research settings, although it is not intended to provide a clinical diagnosis (38, 39).

Although the HADS was originally developed for hospital outpatient populations, it has since been used in broader occupational and health-related survey contexts, including studies assessing mental health among veterinarians in Canada (40). In the present study, HADS was used to estimate the burden of anxiety and depressive symptoms among veterinarians rather than to diagnose anxiety or depressive disorders.

### 2.4 Professional Quality of Life Scale (ProQOL)

ProQOL version 5 was used to assess professional quality of life (25). The ProQOL-5 is a 30-item self-report instrument designed to measure both positive and negative aspects of helping-related work. It includes three 10-item subscales: compassion satisfaction, burnout, and secondary traumatic stress. Compassion satisfaction refers to the pleasure and fulfillment derived from doing one’s work well. Burnout reflects feelings of hopelessness, exhaustion, frustration, and difficulty dealing with work effectively. Secondary traumatic stress refers to work-related distress resulting from secondary exposure to extremely or traumatically stressful events. The wording of selected ProQOL-5 items was adapted to improve their relevance to veterinary practice. References to occupation or helping work were replaced, where appropriate, with terms such as “veterinarian,” “veterinary work,” “cases,” “clients,” “people/animals,” or “those I assist.” In the present study, the ProQOL subscales were analyzed separately as compassion satisfaction, burnout, and secondary traumatic stress. The term “compassion fatigue” was not treated as an independently measured outcome because its conceptual definition remains debated and because the ProQOL operationalizes its negative component through the burnout and secondary traumatic stress subscales.

Each item was scored on a 5-point Likert scale ranging from 1 = never to 5 = very often. Respondents were asked to answer based on their experiences during the previous 30 days. Items 1, 4, 15, 17, and 29 were reverse-scored according to the ProQOL-5 scoring instructions before calculating the burnout subscale score. Subscale scores were calculated by summing the 10 items within each subscale.

### 2.5 Statistical analysis

Data collected through Microsoft Forms were exported as a comma-separated values file and analyzed using R version 4.4.2. No missing values were present because all survey questions were set as mandatory before submission. The normality of the data was determined through the Shapiro–Wilk test. Median and interquartile range (IQR) were calculated for HADS and ProQOL scores. Spearman’s rank correlation analysis was performed between the subscales of ProQOL and HADS. The internal consistency of the HADS and ProQOL subscales was assessed using Cronbach’s alpha coefficients. Mann–Whitney *U* tests were used to compare HADS and ProQOL scores between variables with two categories, whereas Kruskal–Wallis tests were used for variables with three or more categories. Binary logistic regression analyses were performed to assess factors associated with suicidal ideation. Univariable logistic regression analyses were initially conducted for each demographic variable. Subsequently, age group, gender, marital status, and weekly working hours were included simultaneously in a multivariable logistic regression model to estimate adjusted odds ratios (aORs) and 95% confidence intervals (CIs). Due to small sample sizes in some categories, age was categorized as 20–30 years and ≥31 years, and marital status was categorized as single versus married/previously married. Statistical significance was set at *p* < 0.05.

## 3 Results

A total of 456 veterinarians participated in the study. The demographic characteristics of the participants are presented in Table 1. Most respondents were male (*n* = 356, 78.1%), while 100 respondents were female (21.9%). Most participants were aged 20–30 years (*n* = 392, 86.0%), followed by 31–40 years (*n* = 54, 11.8%) and 41 years or older (*n* = 10, 2.2%). Regarding marital status, most respondents were single (*n* = 325, 71.3%), followed by married participants (*n* = 126, 27.6%) and divorced or widowed participants (*n* = 5, 1.1%). More than half of the respondents reported working 0–30 h per week (*n* = 251, 55.0%), while 120 participants worked 31–60 h per week (26.3%) and 85 participants worked 61 or more hours per week (18.6%).

**Table 1:** Demographic characteristics of participating veterinarians in Pakistan (Aug–Nov 2024).

Characteristics	Counts	Percentage
Gender	*N* = 456	—
Male	*n* = 356	78.1%
Female	*n* = 100	21.9%
Age (years)	*N* = 456	—
20–30	*n* = 392	86%
31–40	*n* = 54	11.8%
41+	*n* = 10	2.2%
Marital status	*N* = 456	—
Single	*n* = 325	71.3%
Married	*n* = 126	27.6%
Divorced/Widow	*n* = 5	1.1%
Working hours	*N* = 456	—
0–30	*n* = 251	55%
31–60	*n* = 120	26.3%
61+	*n* = 85	18.6%

Regarding the main type of work, the largest proportion of participants were engaged in mixed animal practice (*n* = 116, 25.4%), followed by farm animal practice (*n* = 86, 18.9%), government service (*n* = 58, 12.7%), industry-related work, including pharmaceutical, feed, and medicine sectors (*n* = 44, 9.7%), small animal practice (*n* = 44, 9.7%), university clinical work (*n* = 36, 7.9%), research (*n* = 24, 5.3%), university non-clinical work (*n* = 22, 4.8%), equine practice (*n* = 10, 2.2%), and other veterinary-related jobs (*n* = 16, 3.5%).

### 3.1 Hospital Anxiety and Depression Scale (HADS)

**Figure 1:**
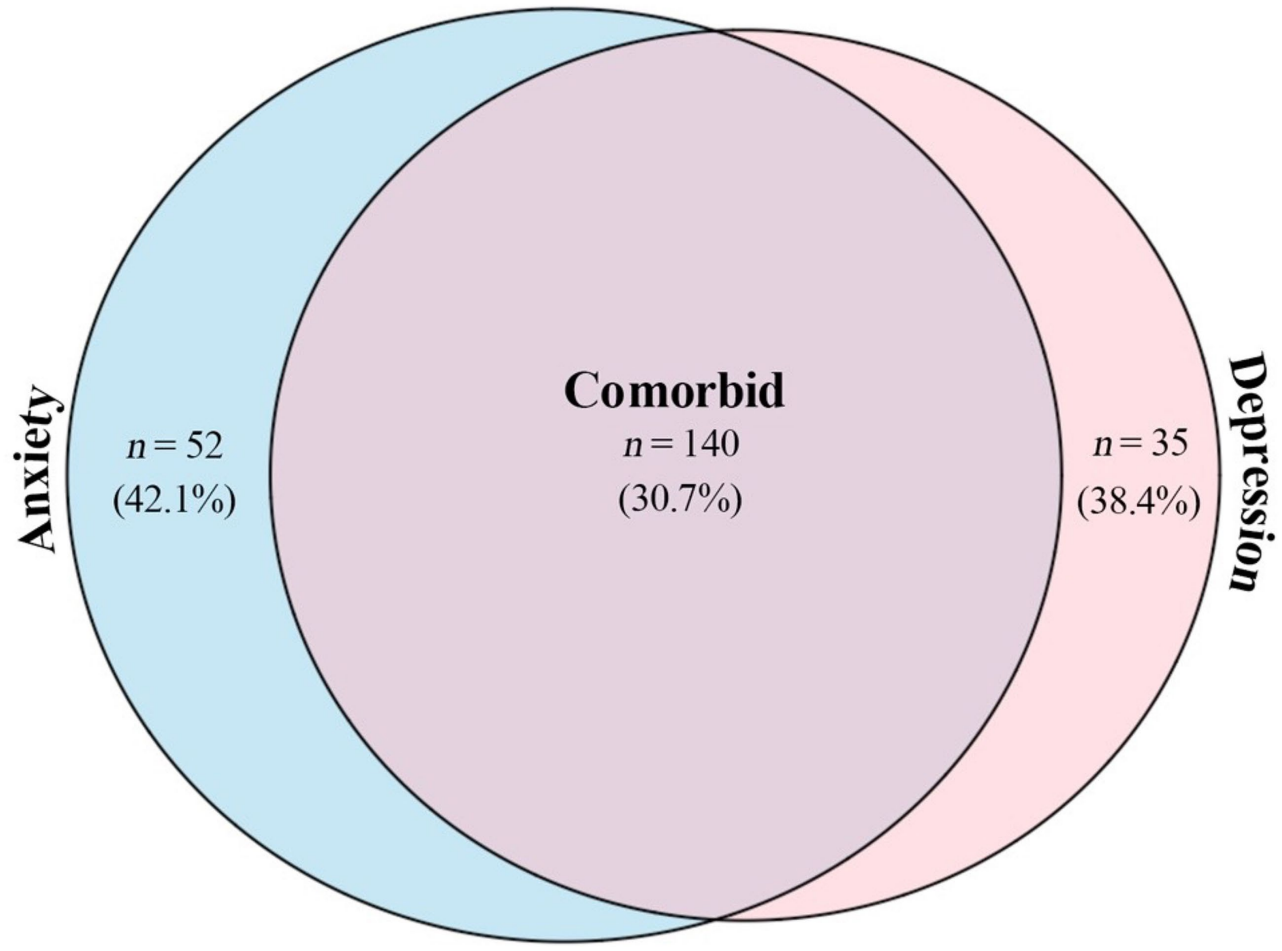
Prevalence of anxiety, comorbid anxiety and depression, and depression. *n* -Anxiety and depression show the participants with HADS ≥11. Percentage of prevalence in parentheses out of the total 456 participants.

The overall median HADS anxiety score was 9.0 (IQR: 6–16). Based on standard HADS cut-offs, 168 respondents (36.8%) were classified as normal, 96 (21.1%) as borderline abnormal, and 192 (42.1%) as abnormal for anxiety symptoms (Table 2). The overall median HADS depression score was also 9.0 (IQR: 5–12). For depression, 182 respondents (39.9%) were classified as normal, 99 (21.7%) as borderline abnormal, and 175 (38.4%) as abnormal. Comorbid abnormal anxiety and depression were observed in 30.7% of participants (Figure 1).

**Table 2:** Hospital Anxiety and Depression Scale (HADS) score results of participating veterinarians in Pakistan (Aug–Nov 2024).

Subscale and participant characteristics	Counts	Median	IQR	0–7	8–10	11–21
	*N* = 456			Normal case (%)	Borderline case (%)	Abnormal case (%)
Subscale and gender						
Anxiety	—	9	6–16	168 (36.8)	96 (21.1)	192 (42.1)
Male	*n* = 356	9	6–13	130 (37.9)	73 (20.5)	153 (43)
Female	*n* = 100	12	6–16	38 (38)	23 (23)	39 (39)
Depression	—	9	5–12	182 (39.9)	99 (21.7)	175 (38.4)
Male	*n* = 356	8	4–12	153 (43)	77 (21.6)	126 (35.4)
Female	*n* = 100	10	6–14	29 (29)	22 (22)	49 (49)
Subscale and age						
Anxiety	—	—	—	—	—	—
20–30	*n* = 392	9	6–14	146 (37.2)	84 (21.4)	162 (41.3)
31–40	*n* = 54	8	5–11	18 (33.3)	9 (16.7)	27 (50)
41+	*n* = 10	7.5	5–9	4 (40)	3 (30)	3 (30)
Depression	—	—	—	—	—	—
20–30	*n* = 392	9	5–13	151 (38.5)	83 (21.2)	158 (40.3)
31–40	*n* = 54	8	4–11	25 (46.3)	14 (25.9)	15 (27.8)
41+	*n* = 10	4.5	3–9.8	6 (60)	2 (20)	2 (20)
Subscale and marital status						
Anxiety	—	—	—	—	—	—
Single	*n* = 325	9	6–14	117 (36)	68 (20.9)	140 (43.1)
Married	*n* = 126	8.5	5–13	49 (38.9)	28 (22.2)	49 (38.9)
Widow/Divorced	*n* = 5	13	8–16	2 (40)	0 (0)	3 (60)
Depression	—	—	—	—	—	—
Single	*n* = 325	9	5–12	128 (39.4)	72 (22.2)	123 (38.5)
Married	*n* = 126	8	4–12	53 (42.1)	27 (21.4)	46 (36.5)
Widow/Divorced	*n* = 5	14	11–14	1 (20)	0 (0)	4 (80)
Subscale and working hours						
Anxiety	—	—	—	—	—	—
0–30	*n* = 251	11	6–15	92 (36.7)	44 (17.5)	115 (45.8)
31–60	*n* = 120	8	7–12	48 (40)	25 (20.8)	47 (39.2)
61+	*n* = 85	8	6–11	28 (32.9)	27 (31.8)	30 (35.3)
Depression	—	—	—	—	—	—
0–30	*n* = 251	9	5–13	100 (39.8)	42 (16.7)	109 (43.4)
31–60	*n* = 120	8	4–12	49 (40.8)	31 (25.8)	40 (33.3)
61+	*n* = 85	8	5–11	33 (38.8)	26 (30.6)	26 (30.6)

*N*, Total number of score results from participating veterinarians; *n*, Total number of participating veterinarians in each category; IQR, Interquartile range.

Comparisons of HADS scores across demographic characteristics are presented in Table 3. Female participants had higher median anxiety scores than male participants [12.0 (IQR: 6–16) versus 9.0 (IQR: 6–13), *p* = 0.017] and higher median depression scores [10.0 (IQR: 6–14) versus 8.0 (IQR: 4–12), *p* = 0.004]. In contrast, the proportion classified in the abnormal anxiety category was slightly higher among males (43%) than among females (39%) (Table 2). This difference reflects the distinction between comparisons of the full score distributions and comparisons based on categorical cut-offs.

**Table 3:** Comparison of anxiety, depression, compassion satisfaction, burnout, and secondary traumatic stress scores across demographic characteristics of veterinary professionals.

Variable	Anxiety median (IQR)	*p*	Depression median (IQR)	*p*	Compassion median (IQR)	*p*	Burnout median (IQR)	*p*	Sec. traumatic stress median (IQR)	*p*
Gender*										
Male	9 (6–13)		8 (4–12)		34 (25–42)		27 (24–30)		25 (20–30)	
Female	12 (6–16)	**0.017**	10 (6–14)	**0.004**	32 (27–41)	0.41	28 (25–30)	**0.031**	28 (23–32)	**0.004**
Age**										
20–30	9 (6–14)	**0.018**	9 (5–13)	0.057	33 (25–42)	**0.031**	27 (24–30)	0.503	25 (20–30)	0.546
31–40	8 (5–11)		8 (4–11)		38 (30–44)		28 (22–30)		28 (20–33)	
41+	7.5 (5–9)		4.5 (3–10)		30 (29–30)		24 (23–27)		22 (20–30)	
Marital status**										
Single	9 (6–14)		9 (5–12)		33 (25–41)		28 (24–30)		25 (20–31)	
Married	8.5 (5–13)	0.12	8 (4–12)	0.282	34 (28–43)	**0.037**	26 (22–29)	0.69	25 (21–30)	0.84
Widow/Divorced	13 (8–16)		14 (11–14)		25 (22–27)		30 (15–32)		22 (22–24)	
Work hours/Week**										
0–30	11 (6–15)	0.15	9 (5–13)	0.407	33 (25–42)	0.627	27 (24–30)	0.661	25 (19–31)	0.124
31–60	8 (7–12)		8 (4–12)		31 (27–41)		27 (24–31)		25 (21–30)	
61+	8 (6–11)		8 (5–11)		35 (25–42)		27 (23–30)		25 (23–32)	

Values are presented as median (IQR). *Mann–Whitney *U* tests were used for variables with two categories, and **Kruskal–Wallis tests were used for variables with three or more categories. Statistically significant *p*-values (*p* < 0.05) are shown in bold.

Anxiety and depression scores did not differ significantly across marital-status or working-hour categories. Although some descriptive variation was observed among these groups, the differences were not statistically significant. Comparisons involving the group aged 41 years or older and the widowed or divorced group should be interpreted cautiously because these categories contained small numbers of participants. Cronbach’s alpha values were 0.83 for the anxiety subscale and 0.76 for the depression subscale.

### 3.2 ProQOL

The overall ProQOL results are presented in Table 4. The median compassion satisfaction score was 33.5 (IQR: 25–42), the median burnout score was 27 (IQR: 24–30), and the median secondary traumatic stress score was 25 (IQR: 20–31).

**Table 4:** The Professional Quality of Life Scale (version 5) results among participating veterinarians.

Factors		*N* (%)	Compassion	Burnout	Sec. traumatic stress
			Median (IQR)	Median (IQR)	Median (IQR)
Gender	Male	356 (78.1)	34 (25–42)	27 (24–30)	25 (20–30)
	Female	100 (21.9)	31.5 (27–41)	28 (25–30)	27.5 (23–32)
Marital status	Single	325 (71.3)	33 (25–41)	28 (24–30)	25 (20–31)
	Married	126 (27.6)	34 (28–43)	26 (22–29)	25 (21–30)
	Widow/Divorced	5 (1.1)	25 (22–27)	30 (15–32)	22 (22–24)
Age	20–30	392 (85.9)	32.5 (25–42)	27 (24–30)	25 (20–30)
	31–40	54 (11.8)	38 (30–44)	27.5 (22–30)	28 (20–33)
	41+	10 (2.2)	30 (29–30)	24 (23–27)	22 (20–30)
Main type of work	Mixed practice	116 (25.4)	33.5 (25–42)	27 (24–30)	25 (21–30)
	Farm animal practice	86 (18.9)	32 (25–40)	27 (22.5–29)	25 (21–31)
	Government	58 (12.7)	35 (30–44)	25 (22–29)	27 (19–32)
	Industry (pharma, feed etc.)	44 (9.7)	29 (23–40)	30.5 (27–33)	28 (25–32)
	Small animal practice	44 (9.7)	31 (25–40)	29 (24–30)	24 (19–31)
	University—clinical	36 (7.8)	29 (25–36)	28 (24–30)	27 (21–37)
	University—non-clinical	22 (4.8)	31 (30–36)	26 (24–29)	27 (22–31)
	Equine practice	10 (2.2)	24 (24–30)	28 (24–28)	18 (15–21)
	Researcher	24 (5.3)	41 (37–42)	24 (23.8–27)	26 (18–30)
	Other	16 (3.5)	26 (24.8–40.5)	29 (21.5–30)	25.5 (18.5–30)
Hours of work per week	0–30	251 (55.0)	33 (25–42)	27 (24–30)	25 (19–31)
	31–60	120 (26.3)	30.5 (27–41)	27 (24–31)	25 (21–30)
	61+	85 (18.6)	35 (25–42)	27 (23–30)	25 (23–32)

*N*, Total number of responses from participating veterinarians. Values are presented as median (IQR). IQR, Interquartile range.

Comparisons of ProQOL scores across demographic characteristics are presented in Table 3. Female participants had higher median secondary traumatic stress scores than male participants [28.0 (IQR: 23–32) versus 25.0 (IQR: 20–30), *p* = 0.004]. No statistically significant gender differences were observed for compassion satisfaction or burnout.

Compassion satisfaction, burnout, and secondary traumatic stress scores did not differ significantly across age, marital status, or working-hour categories. Although some descriptive variation was observed among these groups, the differences were not statistically significant. Descriptive variation was observed across occupational groups, with relatively high compassion satisfaction scores among researchers and government veterinarians and relatively high burnout or secondary traumatic stress scores in some industry and clinical-practice groups. However, these occupational patterns should not be interpreted as statistically significant unless supported by the corresponding group-comparison tests. Comparisons involving the group aged 41 years or older and the widowed or divorced group should be interpreted cautiously because these categories contained small numbers of participants. Cronbach’s alpha values were 0.93 for compassion satisfaction, 0.78 for burnout, and 0.83 for secondary traumatic stress.

### 3.3 Correlation analysis

Spearman’s rank correlation analysis showed that anxiety and depression were strongly positively correlated (*ρ* = 0.71). Burnout was positively correlated with anxiety (*ρ* = 0.31), depression (*ρ* = 0.39), and secondary traumatic stress (*ρ* = 0.39), and negatively correlated with compassion satisfaction (*ρ* = −0.32). Compassion satisfaction was negatively correlated with anxiety (*ρ* = −0.19) and depression (*ρ* = −0.26) but positively correlated with secondary traumatic stress (*ρ* = 0.28). Secondary traumatic stress was also positively correlated with anxiety (*ρ* = 0.28) and depression (*ρ* = 0.24). All reported correlations were statistically significant with *p* < 0.001.

### 3.4 Suicidal ideation

Among the 456 participants, 174 (38.2%) reported that they had felt life was not worth living, 92 (20.2%) reported wishing they were dead, and 112 (24.6%) reported having thought of taking their life even if they would not really do it. Univariable logistic regression results for suicidal ideation-related items are presented in Table 5.

**Table 5:** Univariable logistic regression analysis of suicidal tendencies with different predictors from the participating veterinarians.

Predictors	Have you ever felt that life was not worth living			Have you wished that you were dead			Have you thought of taking your life even if you would not really do it		
	Odds ratios	CI	*p*	Odds ratios	CI	*p*	Odds ratios	CI	*p*
Gender									
Female	—	—	—	—	—	—	—	—	—
Male	0.51	0.32–0.79	**0.003**	0.31	0.19–0.51	0	0.74	0.46–1.24	0.200
Age									
20–30	—	—	—	—	—	—	—	—	—
31+	0.70	0.39–1.22	0.200	1.13	0.57–2.09	0.720	1.03	0.54–1.86	0.900
Marital status									
Single		—	—	—	—	—	—	—	—
Married/Previously married	1	0.66–1.52	0.100	0.91	0.54–1.5	0.700	0.65	0.39–1.05	0.080
Working hours/Week									
0–30	—	—	—	—	—	—	—	—	—
31–60	0.75	0.47–1.17	0.200	0.57	0.32–0.98	**0.050**	0.42	0.23–0.72	**0.002**
61+	0.78	0.47–1.3	0.600	0.26	0.1–0.55	**0.001**	0.48	0.25–0.86	**0.020**

Bold values indicate statistically significant *p*-values (≤0.05).

For the item assessing whether life was perceived as not worth living, male participants had lower odds of reporting this response compared with female participants (OR = 0.51, 95% CI: 0.32–0.79, *p* = 0.003). Age, marital status, and working hours were not significantly associated with this item. For the item assessing whether participants wished they were dead, male participants had lower odds of reporting this response compared with female participants (OR = 0.31, 95% CI: 0.19–0.51, *p* < 0.001). Participants working 31–60 h per week and 61 or more hours per week also had lower odds of reporting this response compared with those working 0–30 h per week. For the third item, “Have you thought of taking your life even if you would not really do it,” working hours were significantly associated with responses. Compared with participants working 0–30 h per week, those working 31–60 h per week and 61 or more hours per week had lower odds of reporting this response. Gender, age, and marital status were not significantly associated with this item.

The results of the multivariable logistic regression analysis are presented in Table 6. After adjustment for the other variables, male participants had 44% lower odds of reporting suicidal ideation than female participants (adjusted OR [aOR] = 0.56, 95% CI: 0.35–0.88, *p* = 0.013). This indicates that the association between gender and suicidal ideation persisted independently of age, marital status, and working hours.

**Table 6:** Multivariable logistic regression analysis of demographic factors associated with suicidal ideation among veterinarians.

Variable	Adjusted OR (95% CI)	*p*-value
Gender		
Female (ref)	—	—
Male	0.56 (0.35–0.88)	0.013
Age		
20–30 (ref)	—	—
31+	1.01 (0.51–1.99)	0.986
Marital status		
Single (ref)	—	—
Married/Previously married	0.98 (0.59–1.65)	0.952
Working hours/Week		
0–30 (ref)	—	—
31–60	0.59 (0.36–0.96)	0.036
61+	0.85 (0.50–1.42)	0.523

Working hours also remained independently associated with suicidal ideation. Compared with participants working 0–30 h per week, those working 31–60 h per week had 41% lower adjusted odds of reporting suicidal ideation (aOR = 0.59, 95% CI: 0.36–0.96, *p* = 0.036). In contrast, the association for participants working 61 or more hours per week was attenuated after adjustment and was no longer statistically significant (aOR = 0.85, 95% CI: 0.50–1.42, *p* = 0.523). This suggests that the apparent associations observed for longer working hours in some of the univariable analyses may have been partly influenced by differences in gender, age, or marital status.

Age was not independently associated with suicidal ideation: participants aged 31 years or older had odds similar to those aged 20–30 years (aOR = 1.01, 95% CI: 0.51–1.99, *p* = 0.986). Marital status was also not independently associated with the outcome, with married or previously married participants having odds similar to single participants (aOR = 0.98, 95% CI: 0.59–1.65, *p* = 0.952). Overall, female participants and those working 0–30 h per week had comparatively higher odds of suicidal ideation within this sample. Cronbach’s alpha for the three suicidal ideation-related items was 0.66, indicating questionable to acceptable internal consistency.

## 4 Discussion

The principle that there is “no health without mental health” has been emphasized by the WHO and other public health organizations, including the Royal College of Psychiatrists and the Pan American Health Organization (41, 42). Mental health and well-being are important public health concerns because anxiety, depression, and stress can cause reduced productivity, poorer quality of care, lower patient satisfaction, and increased risk of errors in healthcare settings (43, 44). Veterinarian mental health has been reported as an area of concern in several countries, with studies from the United Kingdom, the United States, Canada, New Zealand, and Australia reporting high levels of anxiety, depression, burnout, secondary traumatic stress, and suicidal ideation (45–48). To our knowledge, this is among the first studies to assess mental health, suicidal ideation, and professional quality of life among veterinarians in Pakistan.

In the present study, a substantial proportion of participating veterinarians were classified in the abnormal category for both HADS-A and HADS-D, with percentages of 42.1 and 38.4%, respectively. These findings are consistent with previous studies from the United Kingdom (48), Austria (49), and Canada (40), where veterinarians have been reported to experience high levels of anxiety, depression, burnout, secondary traumatic stress, and poorer mental well-being compared with general population estimates. Previous research has found that a toxic team environment is associated with greater burnout and poorer job satisfaction (50). Although the proportion classified in the abnormal anxiety category was marginally higher among males, female veterinarians had significantly higher median anxiety scores. This apparent discrepancy may reflect the loss of information that occurs when continuous scores are divided into categories using fixed cut-off values. Female veterinarians had significantly higher median depression scores and a descriptively higher prevalence of abnormal depression scores than male veterinarians. This finding is consistent with previous evidence of gender differences in mental-health outcomes within the veterinary profession (48, 51). A high proportion of participants in the youngest age group were classified as having abnormal anxiety or depression, which may reflect early-career stressors such as job insecurity, financial instability, and the challenges of transitioning from academic to professional environments. However, differences across age categories were not statistically significant, and interpretation is limited because 86% of the sample was aged 20–30 years. One possible explanation for the high anxiety and depression scores may be the broader context of unemployment and underemployment in Pakistan (52). In addition, Pakistani veterinarians may encounter occupational stressors, including concerns related to zoonotic exposure and workplace conditions (9). Participants working 0–30 h per week had the highest descriptive proportions of abnormal anxiety and depression. This may reflect the potential relationship between reduced working hours and lower income in developing countries, as a previous study has reported an association between lower income and higher depression levels in Pakistan (53).

The ProQOL findings suggest that professional well-being in this population may be associated with both personal and work-related factors. The slightly higher secondary traumatic stress observed among female veterinarians may indicate greater emotional burden, possibly related to differences in workplace experiences, caregiving expectations, and client interactions (48, 53, 54). This finding is particularly important because secondary traumatic stress can contribute to emotional exhaustion and may affect both personal well-being and professional performance. Gender differences in professional quality of life have also been reported in other healthcare professions, including emergency medicine (54). In a recent study, approximately 23% of veterinarians were assigned to a high emotional-risk profile, while 50.4% showed substantial emotional exhaustion (55). Descriptively, veterinarians working in the pharmaceutical industry had the highest burnout scores, whereas university clinical veterinarians had the highest secondary traumatic stress scores. However, differences across occupational groups were not statistically significant. University clinical veterinarians may experience a combination of clinical responsibilities, teaching duties, administrative demands, and responsibility for complex referral cases. These potential explanations were not measured in the present study and require further investigation. Evidence specifically concerning veterinarians in pharmaceutical marketing is limited; sales-related roles in the pharmaceutical sector are often characterized by performance targets, extensive travel, and work–life imbalance, which have been associated with occupational stress and burnout among medical representatives (56). The result of clinical staff burnout is alarming, according to previous studies regarding burnout in veterinary surgeons (57) and anesthetists (58). Furthermore, exposure to ethically and emotionally challenging procedures, such as euthanasia, is the probable reason for burnout and suicidal ideation in veterinary surgeons and physicians (32). Researchers and government-employed veterinarians reported higher compassion satisfaction scores and slightly lower levels of burnout and secondary traumatic stress. This may be partly explained by greater job security, interest, working income, and more predictable working conditions compared with other employment sectors (59). Veterinarians working longer hours reported higher levels of burnout and secondary traumatic stress, consistent with findings from New Zealand (47). In another study, using data from 5,786 associate veterinarians, it was estimated that burnout costs the United States veterinary industry approximately United States $1-2 billion annually through turnover and reduced working hours (60). Although the differences observed in our study were not pronounced, the trend suggests that extended working hours may contribute to increased occupational stress among veterinarians. In developing countries such as Pakistan, longer working hours may be associated with higher income or greater job stability, which could partly explain why burnout and secondary traumatic stress did not differ significantly across working-hour groups in our study.

Veterinarians have been reported to be at higher risk of suicidal ideation than the general population (48). The present study findings suggest that suicidal ideation among participating veterinarians is potentially associated with both gender and occupational workload. Male veterinarians had lower odds of reporting suicidal ideation compared with female veterinarians, suggesting a greater burden of suicidal ideation-related outcomes among female respondents. These findings are consistent with previous veterinary mental health studies reporting poorer mental health indicators among female veterinarians, including higher anxiety, depression, burnout, and secondary traumatic stress scores (48, 54, 61). In the current study, female participants also had significantly higher secondary traumatic stress scores. Although this finding may indicate a broader pattern of psychological and occupational distress, the present analysis cannot determine whether secondary traumatic stress contributed to suicidal ideation-related outcomes. Working hours were also associated with suicidal ideation, with veterinarians working 31–60 h per week having lower odds of suicidal ideation compared with those working 0–30 h per week. Although fewer working hours may appear protective, in this context they may also reflect underemployment, job insecurity, reduced income, or lower professional engagement, all of which may contribute to psychological distress. Low income and financial insecurity have been associated with poorer mental health outcomes, including depression and anxiety, and may be particularly relevant for early-career professionals in settings with limited employment opportunities (62). Salary, emotional support, and mental well-being are important factors associated with veterinarians’ professional quality of life (61).

This study has limitations that should be considered when interpreting the findings. The data were collected using self-report questionnaires, which may be affected by recall bias and by differences in individuals’ willingness to disclose psychological distress. Participants’ income was not assessed in this study, which represents an important limitation. Income and financial security may influence professional quality of life, including compassion satisfaction, burnout, and secondary traumatic stress. The wording of the ProQOL-5 was only partially adapted to veterinary practice. Although several items referred specifically to veterinarians, animals, clients, cases, or veterinary workload, some retained human-service terms such as “people,” “person,” and “trauma victims.” These terms may have been interpreted differently by respondents, including as references to animals, animal owners, clients, colleagues, or combinations of these groups. Although ProQOL-5 is widely used, previous psychometric evaluations have raised concerns regarding the measurement adequacy of the original burnout and secondary traumatic stress subscales and the stability of its proposed three-factor structure (63, 64). Participation in this study was voluntary; veterinarians with strong interest in mental health or those experiencing distress may have been more inclined to respond, whereas others may have ignored the survey due to lack of time, limited internet access, survey fatigue, or discomfort with the topic. The sample was predominantly composed of veterinarians aged 20–30 years, with limited representation of older age groups. Therefore, the findings may not be generalizable to mid-career or older veterinarians. In addition, online convenience sampling may have introduced age-related selection bias, and national data were unavailable to assess whether the sample reflected the age distribution of veterinarians in Pakistan. Participants working 31–60 h per week had lower adjusted odds of suicidal ideation than those working 0–30 h per week. However, working hours and suicidal ideation were assessed at the same time, and the cross-sectional design does not permit the determination of the temporal direction of this association. Cross-sectional analyses may be susceptible to reverse causality when it is unclear whether the exposure preceded the outcome. Thus, veterinarians experiencing psychological distress may have reduced their working hours because of their mental or physical health. Alternatively, fewer working hours could reflect underemployment, job insecurity, reduced income, or limited professional opportunities. Although HADS and ProQOL-5 are widely used screening instruments, they have not been specifically validated among Pakistani veterinarians. Therefore, the findings should be interpreted as indicators of psychological distress and professional quality of life rather than clinical diagnoses.

## 5 Conclusion

This is among the first studies to assess mental health and professional well-being among veterinarians in Pakistan, highlighting a substantial burden of psychological distress within the profession. High levels of anxiety, depression, suicidal ideation-related indicators, burnout, and secondary traumatic stress were observed in veterinarians working in different fields. Female veterinarians had significantly higher anxiety, depression, and secondary traumatic stress scores and greater odds of some suicidal ideation-related outcomes than male veterinarians. The association between reduced working hours and poorer mental health outcomes is especially important in the Pakistani context, where fewer working hours may reflect underemployment, low income, or limited professional opportunities rather than improved work-life balance; however, these potential explanations were not directly measured and require further investigation. Differences observed across age and occupational groups were largely descriptive and should be interpreted cautiously because most were not statistically significant, and some subgroups were small. Overall, the study highlights an urgent need for targeted mental health interventions, improved workplace support systems, and policy-level changes to reduce occupational stress and enhance well-being. Addressing these challenges is essential not only for the health of veterinary professionals but also for sustaining the quality and effectiveness of veterinary services. Future longitudinal and multicenter studies are needed to confirm these findings, identify causal pathways, and guide targeted interventions to improve the well-being and sustainability of the veterinary workforce in Pakistan.

## Data Availability

The original contributions presented in the study are included in the article/supplementary material, further inquiries can be directed to the corresponding author.

## Footnotes

1
https://pvmc.gov.pk/
